# Evaluation of functional capacity and risk of depression in older patients with cancer as part of the comprehensive geriatric assessment

**DOI:** 10.3389/fnagi.2025.1595336

**Published:** 2025-05-16

**Authors:** Wiesław Fidecki, Katarzyna Przylepa, Marek Wytrzyszczewski, Mariusz Wysokiński

**Affiliations:** ^1^Laboratory of Clinical Skills Chair of Development in Nursing, Faculty of Health Sciences, Medical University of Lublin, Lublin, Poland; ^2^Faculty of Medical Sciences, Medical University of Lublin, Lublin, Poland; ^3^Faculty of Health Sciences, Department of Basic Nursing Chair of Development in Nursing, Medical University of Lublin, Lublin, Poland

**Keywords:** functional capacity, depression, cancer patients, older adults, NOSGER

## Abstract

**Background:**

The patient’s somatic health status plays an important role in the factors predisposing to the development of depression. Any disease, especially a chronic one, often associated with pain, may contribute to the development of depression. The aim of the study was to assess the impact of functional capacity on the occurrence of depression among elderly cancer patients.

**Methods:**

The work used the diagnostic survey method and survey technique research tool consisted of standardized questionnaires: the Nurses’ Observation Scale for Geriatric Patients (NOSGER), the Barthel scale, and the Geriatric Depression Scale (GDS). The authors conducted the study among 110 older adults patients with hospitalized at the Lublin Region Oncology Center in 2022.

**Results:**

When assessing patients using the NOSGER scale, the average result for the entire group was 45.98 ± 12.58 points. The examined people functioned best in terms of memory - average of 5.99 ± 1.32 points. The worst functioning was in the instrumental activities of everyday life - average 9.16 ± 3.61 points. In the assessment of the functional status using the Barthel scale, 54.54% of the respondents were classified as light. Based on the assessment of patients using the GDS scale, it was found that as many as 66.36% of the respondents did not experience symptoms of depression. The regression model showed that the NOSGER scale was a statistically significant variable explaining the geriatric depression scale score in the study group (*p* < 0.001).

**Conclusion:**

Most respondents were fully functional. The surveyed seniors suffering from cancer had the greatest difficulties in the areas of moods and emotions as well as instrumental activities of everyday life. The occurrence of depression symptoms was found in over 30.00% of respondents. It is advisable to use research tools that take into account a wider range of aspects when assessing the impact of functional capacity on the possibility of depression among geriatric patients with cancer.

## 1 Introduction

The systematic increase in the number of older adults around the world is also accompanied by a systematic increase in the incidence of cancer in this population (Goldzweig et al., 2022; Gosney, 2005).

Due to the above, many health care organizational systems are or will soon be faced with the dilemma of optimal use of their resources, especially in the context of multidisciplinary cooperation, i.e., geriatricians, oncologists, nurses and pharmacists (Herledan et al., 2023). Seniors diagnosed with cancer may have a negative impact on their physical fitness, cognitive processes, mental and emotional state. These limitations may not only be the result of cancer, but also result from changes occurring in the human body during old age, or from multi-morbidities common in this age group. Therefore, in the care of older adults with cancer, it is necessary to use tools that enable monitoring and assessment of the dynamics of these processes (Loh and Mohile, 2023).

One particular area that requires monitoring is the area of functional capacity. In older people, it depends not only on the aging process, lifestyle, environmental, social and psychological factors, as well as co-existing chronic diseases (Biercewicz, 2021). The limitation of functional capacity itself may, on the one hand, be a symptom of the disease, and on the other hand, lead to many unfavorable phenomena, e.g., the occurrence of pressure sores, feelings of loneliness, or the intensification of pain, emotional disorders, or even depression (Fettes et al., 2021). Depression in the group of seniors, especially those over 70 years of age, is rarely diagnosed (Goldzweig et al., 2022), despite the fact that nearly 40% of them have clinically significant anxiety (Clausing et al., 2023). Depression among seniors may have various pathogenesis. It may develop as a response to a diagnosis or as a result of disease symptoms or treatment. Among geriatric patients, it is most often chronic (Uysal and Poyraz, 2023). It may also arise as a result of limited functional capacity of seniors. There are few studies analyzing how the limitation of functional capacity affects the development of depression, especially in geriatric patients with cancer. Both in relation to the Polish and international population. The presented study tries to fill this gap In this approach, the assessment of the functional status of older adults with cancer can also be used as a prognostic element (Kitamura et al., 2020).

The aim of the study was to assess the impact of functional capacity on the occurrence of depression among older adults cancer patients.

The main problem was related to the question what is the impact of functional capacity on the occurrence of depression among older adults with cancer? It was specified by an attempt to determine the impact of sociodemographic variables on functional capacity and the occurrence of depression in the studied group of patients.

## 2 Materials and methods

### 2.1 Research organization and participants

The study was conducted in 2022 among a group of 110 older adults with cancer hospitalized at the Lublin Region Oncology Center in 2022. Because these are pilot research, no attempt was made to determine the sample size. All persons meeting the inclusion criteria were included. The research material was collected by the authors of the work.

The inclusion criteria for the study are:

1. Age over 60.
2. Diagnosis of cancer confirmed by histopathological examination, regardless of whether treatment has been initiated or not.
3. Consent of respondents to participate in the study.

The criteria for exclusion from the research are:

1. Age below 60 years.
2. Lack of histopathological confirmation of the diagnosis of neoplastic disease.
3. Respondents’ lack of consent to participate in the research.

### 2.2 Method

The study used the diagnostic survey method, survey technique. The study used standardized NOSGER, Barthel and Geriatric Depression Scale questionnaires.

The NOSGER scale was developed by Spiegel et al. in 1991 (Spiegel et al., 1991). It consists of 30 statements representing 6 areas: memory (P); instrumental activities of daily living (IADL), activities of daily living (ADL), moods and emotions (ME), social behaviors (S), disruptive behaviors (Z). A person tested in each issue can score from 1 to 5 points, which allows for a score ranging from 30 to 150 points. The lower the score, the better the patient’s condition (Fidecki et al., 2020). The NOSGER scale does not have specific score ranges for interpretation during assessment. More important than the total number of points obtained is their distribution in individual component areas. This pattern provides information about deficits in particular areas and may suggest a given pathology (Fidecki et al., 2021). There is also no clear interpretation of the results, but the authors of the scale propose to consider the following scores as the upper limit of normal values: 10 points memory, 11 points IADL, 8 points mood, 10 points social behavior, 7 points disruptive behavior. However, threshold values for the ADL area have not been precisely defined (Bläsi et al., 2005). In 2020, it was validated into Polish (Fidecki et al., 2020). The Cronbach’s alpha coefficient value of the NOSGER scale in the study group was 0.877.

The study also used the Barthel scale. It was developed by Mahoney and Barthel in 1965. In Poland, it is commonly used to assess the patient’s level of independence in everyday activities. It evaluates 10 activities and assigns them appropriate point values. Obtaining 100 points means full independence. Obtaining from 86 to 99 points. (category I) means a “light patient” requiring little assistance, from 21 to 85 points (category II) means a “medium patient” requiring assistance in most activities, and from 0 to 20 points (category III) means a “severe patient” requiring assistance in all activities (Opara et al., 2015). The Cronbach’s alpha coefficient value of the Barthel scale in the study group was 0.799.

The Geriatric Depression Scale (GDS) was used as a research tool. This scale was developed in 1983 by Yesavage et al. (1983) as a screening tool to assess the intensity of depression symptoms in older adults. It consists of 30 short questions with two answer options (yes/no). As a standard, GDS – LF (long form) scoring is used, according to which a result from 0 to 10 points means no depression, from 11 to 20 points indicates a slight depression, and a result from 21 to 30 points suggests the presence of deep depression (Figueiredo-Duarte et al., 2021). In 2002, it was validated into Polish (Bidzan et al., 2002). The Cronbach’s alpha coefficient value of the GDS scale in the study group was 0.855.

The selection of tools was deliberate. The NOSGER scale is a new tool in Poland that allows for a comprehensive assessment of functional capacity, while the Barthel scale is currently the most commonly used tool for its assessment in Poland. On the other hand, the GDS scale, due to its structure, is a tool that is easy to use and does not burden the examined senior. The NOSGER scale also allows to avoid the issue of underestimating the symptoms of depression or inadvertently attributing symptoms of a somatic disease to depression (Giannouli, 2017).

### 2.3 Statistical analysis

The statistical analysis and database were performed using Statistica 10.0 software (StatSoft, Poland).

The results obtained in the analysis of quantitative variables were presented using the mean, median and standard deviation values, and in the area of qualitative variables using the frequency and percentage. The normality of the distribution of variables was tested using the Shapiro–Wilk normality test. Differences between groups were assessed using the Mann–Whitney test for two groups, and in the case of three or more groups, the Kruskal-Wallis test was used to perform an ANOVA (with the post-hoc RIR Turkey test). If the requirements for its use were not met, the Kruskal-Wallis test was used. A regression model was also created. In the statistical analysis, a significance level of *p* < 0.05 was adopted to determine the occurrence of statistically significant dependencies or differences.

### 2.4 Ethics statement

The consent to conduct the research was obtained from the Bioethics Committee of the Medical University of Lublin (Resolution No. EC-0254/45/02/2023).

## 3 Results

### 3.1 Sociodemographic analysis of the study group

The study involved 110 older adults diagnosed with cancer, most of whom were men (54.55%). The average age of the respondents was 69.67 years (SD = 6.38). The largest group were people aged 66–74 (46.36%), married (65.46%), declaring primary education (33.64%), living with their family (80.91%). The average duration of the disease was estimated by respondents at 2.53 years (SD = 2.23). The characteristics of the study group are presented in Table 1.

**Table 1:** Sociodemographic analysis of the study group.

Variable		Number (N)	(%)
Gender	Female	50	45.45
	Male	60	54.55
Age	60–65 years old	33	30.00
	66–74 years old	51	46.36
	75–88 years old	26	23.64
Marital status	Single	38	34.54
	Married	72	65.46
Education	Elementary	37	33.64
	Vocational	28	25.45
	Secondary	34	30.91
	Higher	11	10.00
Lives	With family	89	80.91
	Alone	21	19.09

### 3.2 NOSGER scale

The analysis of the study results showed that the average sum of points obtained on the NOSGER scale by the respondents was 45.98 ± 12.58. The subjects functioned best in the area of memory (M) (average 5.99), and the worst in instrumental activities of daily living (IADL) (average 9.16). There was a statistically significant difference between women and men in the average number of points obtained in the areas of instrumental activities of daily living (IADL) (*Z* = 3.365, *p* = 0.000), moods and emotions (ME) (*Z* = 2.497, *p* = 0.012), behaviors social (S) (*Z* = 3.320, *p* = 0.000) of the NOSGER scale. The tested group of women obtained a statistically significantly (*Z* = 3.025, *p* = 0.002) higher result (*M* = 50.16) compared to the male population (M = 42.50). Moreover, there was a statistical difference in the average number of points obtained in the IADL area (*H* = 8.757, *p* = 0.012), S (*H* = 6.418, *p* = 0.040) and the total score (*H* = 7.583, *p* = 0.022) depending on age subjects. In all areas except disruptive behavior, people aged 60–65 functioned better. Analyzing the study group in terms of marital status, a statistically significant difference was found in all areas, except for disruptive behaviors (Z). According to the NOSGER scale, married people had better functional efficiency in the study group. There was also a statistically significant relationship between the respondents depending on the level of education in the areas of IADL (*H* = 9.706, *p* = 0.013), ADL (*H* = 8.062, *p* = 0.044) and the overall result (*H* = 9.631, *p* = 0.022). People declaring higher education had better functional efficiency in all areas. There was also a statistically significant relationship between people living with family members and people living alone in the domains of IADL (*Z* = 2.633, *p* = 0.008), ME (*Z* = 2.918, *p* = 0.003), S (*Z* = 3.077, *p* = 0.002) and the result general (*Z* = 2.858, *p* = 0.004). In all areas, people living alone showed a better level of functioning (Table 2).

**Table 2:** Results of the NOSGER scale in relation to the sociodemographic characteristics of the respondents.

Variable		NOSGER						
		P (M)	IADL (M)	ADL (M)	ME (M)	S (M)	Z (M)	Total (M)
Gender	Female	6.22	10.34	7.46	10.14	9.02	6.98	50.16
	Male	5.80	8.18	6.98	8.18	6.87	6.48	42.50
Statistical analysis		*Z* = 1.354 *p* = 0.175	*Z* = 3.365 *P* = 0.000	*H* = 0.520 *p* = 0.603	*Z* = 2.497 *P* = 0.012	*Z* = 3.320 *P* = 0.000	Z = 1.794 *p* = 0.072	*Z* = 3.025 *P* = 0.002
Age	60–65 years old	5.61	8.00	6.52	8.27	7.09	6.91	42.39
	66–74 years old	6.08	9.06	7.22	9.22	7.65	6.51	45.73
	75–88 years old	6.31	10.85	8.04	9.81	9.19	6.85	51.04
Statistical analysis		*H* = 5.874 *p* = 0.053	*H* = 8.757 P = 0.012	*H* = 5.561 *p* = 0.062	*H* = 1.095 *p* = 0.578	*H* = 6.418 *P* = 0.040	*H* = 1.494 *p* = 0.472	*H* = 7.583 *p* = 0.022
Marital status	Single	6.39	11.00	7.74	10.63	9.39	6.82	51.97
	Married	5.78	8.19	6.92	8.25	7.03	6.65	42.82
Statistical analysis		*Z* = 2.550 *P* = 0.010	*Z* = 4.308 *P* = 0.000	*Z* = 2.474 *P* = 0.013	*Z* = 2.913 *P* = 0.003	*Z* = 3.518 *p* = 0.000	*Z* = 0.619 *p* = 0.535	*Z* = 4/303 *p* = 0.000
Education	Elementary	6.46	10.05	7.30	9.70	8.27	6.84	48.62
	Vocational	5.82	7.89	6.64	8.64	7.04	6.68	42.71
	Secondary	5.85	9.82	7.94	9.23	8.52	6.64	48.02
	Higher	5.27	7.36	6.00	7.54	6.36	6.54	39.09
Statistical analysis		*H* = 5.939 *p* = 0.114	*H* = 9.706 *P* = 0.013	*H* = 8.062 *P* = 0.044	*H* = 2.452 *p* = 0.484	*H* = 5.623 *p* = 0.131	*H* = 0.540 *p* = 0.910	*H* = 9.631 *P* = 0.022
Lives	With family	6.14	10.76	7.62	11.14	10.14	6.95	52.76
	Alone	5.96	8.79	7.10	8.58	7.30	6.65	44.38
Statistical analysis		*Z* = 0.987 *p* = 0.323	*Z* = 2.633 *P* = 0.008	*Z* = 1.469 *p* = 0.141	*Z* = 2.918 *P* = 0.003	*Z* = 3.077 *P* = 0.002	*Z* = 1.059 *p* = 0.289	*Z* = 2.858 *P* = 0.004
Total NOSGER		5.99	9.16	7.20	9.07	7.85	6.71	45.98

Memory (M); instrumental activities of daily living (IADL), activities of daily living (ADL), mood and emotions (ME), social behaviors (S), disruptive behaviors (Z).

*M*, mean; *H*, Kruskal – Wallis test; *Z*, Mann – Whitney test; *p*, statistical significance.

### 3.3 Barthel scale

In the studied group of older adults with cancer, the majority of 54.54% (*N* = 60) were classified as mild patients. A large group, i.e., 43.64% (*N* = 48) of the respondents, was classified as moderately severe, and only 1.81% (*N* = 2). The average number of points obtained by the respondents was 86.45 (SD = 14.90). Using Speraman’s rank correlation, it was found that an increase in the age of the subjects leads to a decrease in their functional capacity (*R* = −0.227, *p* = 0.016). In the case of the Barthel scale, a statistically significant difference occurred only in relation to the marital status of the respondents (*Z* = − 2.574, *p* = 0.010). Married people showed better functional capacity (Table 3).

**Table 3:** Barthel scale results in relation to the sociodemographic characteristics of the respondents.

Variable		Barthel			Statistical analysis
		*M*	Me	SD	
Gender	Female	85.60	87.50	16.43	Z = −0.374 *p* = 0.707
	Male	87.16	90.00	13.60	
Age	60–65 years old	88.18	90.00	14.72	H = 5.490 *p* = 0.064
	66–74 years old	87.84	90.00	13.12	
	75–88 years old	81.53	80.00	17.70	
Marital status	Single	82.36	82.50	16.22	Z = − 2.574 P = 0.010
	Married	88.61	90.00	13.79	
Education	Elementary	83.78	85.00	12.82	H = 6.939 *p* = 0.073
	Vocational	88.21	90.00	16.45	
	Secondary	85.88	90.00	17.12	
	Higher	92.72	95	7.19	
Lives	With family	86.62	90.00	15.77	Z = −1.150 *p* = 0.250
	Alone	85/71	80.00	10.75	

M, mean; Me, median, SD, standard deviation; H, Kruskal – Wallis test; Z, Mann – Whitney test, *p*, statistical significance.

### 3.4 GDS scale

In the study group, 66.36% (*N* = 73) of respondents were not at risk of developing depression based on the GDS scale. 25.46% (*N* = 28) of respondents were classified as mild depression, and 8.18% (*N* = 9) as severe depression. The average score of the respondents was 8.68 (SD = 5.83). Women, people who were not in a relationship and those living alone were more likely to suffer from depressive symptomatology (Table 4).

**Table 4:** The results of the GDS scale in relation to the sociodemographic characteristics of the respondents.

Variable		GDS			Statistical analysis
		*M*	Me	SD	
Gender	Female	11.14	9.00	6.86	*Z* = 3.530 *P* = 0.000
	Male	6.63	6.00	3.80	
Age	60–65 years old	7.848	7.00	4.51	*H* = 1.777 *p* = 0.411
	66–74 years old	8.58	7.00	6.30	
	75–88 years old	9.92	8.00	6.35	
Marital status	Single	12.28	10.50	6.54	*Z* = 4.459 *P* = 0.000
	Married	6.77	6.00	4.39	
Education	Elementary	9.97	7.00	7.08	*H* = 4.817 *p* = 0.185
	Vocational	8.10	7.50	4.39	
	Secondary	8.91	8.00	5.74	
	Higher	5.09	5.00	2.84	
Lives	With family	7.53	7.00	5.08	*Z* = 3.934 *P* = 0.000
	Alone	13.52	12.00	6.46	

*M*, mean; Me, median, SD, standard deviation; *H*, Kruskal – Wallis test; *Z*, Mann – Whitney test, *p*, statistical significance.

### 3.5 Functional capacity and the occurrence of depressive symptomatology among older adults patients

In order to determine the impact of functional capacity on the occurrence of depressive symptomatology among older adults cancer patients, a regression model was developed that explains 69.8% of the variance. In this model, a statistically significant variable explaining the Geriatric Depression Scale (G) score is the NOSGER scale (higher NOSGER scores correspond to higher G scale scores). However, the Barthel scale score is no longer statistically significant (in other words: the Barthel scale score does not have a statistically significant impact on the better prediction of the G score) (Table 5).

**Table 5:** Functional capacity and the occurrence of depressive symptomatology among older adults patients.

	R = 0.839, R2 = 0.704, R2score = 0.698, *F* = 121.123, *p* < 0.001				
	*b*	−95% CI	+95% CI	*p*-value	Stand. *b*
	−16.055	−24.817	−7.293	<0.001	
NOSGER	0.448	0.383	0.512	<0.001	0.880
Barthel	0.050	−0.027	0.127	0.201	0.082

## 4 Discussion

The study showed that the correct assessment of the impact of functional capacity on the occurrence of depressive symptomatology among geriatric patients with cancer requires the use of tools that take into account its broader aspect and not only elements related to physical capacity.

Functional fitness from the point of view of seniors and the health care system is an extremely important issue (Stauder et al., 2010). On an individual level, it guarantees the individual independence in performing necessary daily life activities. In the institutional dimension, its limitation or lack may determine the need for hospitalization. Its limitation or lack leads to a gradual deterioration of the health of seniors. There are accumulating health problems, for example decreased immunity, constipation, increased risk of falls, risk of developing pressure sores and many others. It also leaves its mark on the mental sphere, often leading to the development of depression. In this approach, the assessment of functional capacity can also be treated as a predictive tool predicting the possibility of occurrence of serious symptoms in older adults with cancer (Seow et al., 2021) or mortality after diagnosis of cancer (Jensen et al., 2024).

To the best of the authors’ knowledge, this is the first study of this type in Poland and one of the few in the world trying to answer the question related to determining the impact of functional capacity on the risk of developing depression in geriatric patients with cancer. Due to the above, it is not possible to fully compare the results obtained both to the Polish population and from other countries, despite the use of research tools used by the researcher from an international perspective. Most studies assess functional capacity through the prism of selected phenomena characteristic of old age, for example malnutrition (van den Broeke et al., 2018; Leo et al., 2023; Overcash et al., 2022; Overcash et al., 2023), depression and anxiety (Evangelou et al., 2023; Zhang et al., 2022). Loneliness (Zhang et al., 2023), its impact on caregivers of older adults cancer patients (Overcash et al., 2022), or whether it is associated with the occurrence of violence against seniors (da Silva et al., 2023; Atim et al., 2023) or survival during the COVID-19 pandemic (Frigotto et al., 2023). Other studies, however, analyze the impact of depression on functional capacity. Studies conducted among patients with colorectal cancer awaiting surgery confirmed worse functional capacity in patients with depression (Barrett Bernstein et al., 2019). The impact of depression on functional capacity was also confirmed among cancer patients in Vietnam (Vu et al., 2023) and older adults requiring long-term care in Turkey (Güdük, 2023). It should be noted, however, that most studies examining the relationship between functional capacity and depression are conducted on age groups other than seniors. This may be due to downplaying the possibility of this problem occurring in this age group or diagnostic difficulties related to the occurrence of the so-called masked depression.

The respondents obtained an average result on the NOSGER scale of 45.98 ± 12.58 points. It should be interpreted as satisfactory, especially in the context of the studied group. There are no Polish studies allowing comparison with another group of geriatric cancer patients. However, compared to other groups of patients, it should be stated that the functional efficiency of these patients is better than that of patients with neurological diseases (Biercewicz et al., 2018), internal medicine diseases (Fidecki et al., 2018), and, above all, seniors with dementia (Fidecki et al., 2022) and even those staying at home (Głowacka et al., 2017). Similar results were obtained only by seniors aged 59–75, staying in social welfare homes (Ulatowska et al., 2020). This result is also better than among Turkish seniors with adaptation problems (Kaplan and Keser, 2021). This may be due to the fact that cancer diagnoses take place at an increasingly earlier stage of their development, and particular types of treatment require seniors to be in good enough general condition to have a chance of surviving the treatment.

The best average result was obtained in the field of M (Memory). Other groups of Polish geriatric patients, for example those with neurological diseases, achieved the best results in the domain of disruptive behavior (Z) (Biercewicz, 2021), similarly for seniors staying in social welfare homes (Ulatowska et al., 2020), or with dementia (Fidecki et al., 2022).

The worst in the areas of IADL (instrumental activities of daily living) points and ME (moods and emotions). The obtained results are still better than in the group of geriatric patients with neurological diseases (Biercewicz, 2021) or staying in social welfare homes (Ulatowska et al., 2020) or with dementia (Fidecki et al., 2022). However, Polish seniors staying at home achieved the worst results in the field of social behavior (Głowacka et al., 2017). Unfortunately, this may prove that these people have difficult social contacts, which may, among other things, result from their limitations in functional capacity.

The conducted research showed a relationship between the sociodemographic characteristics of the subjects and the assessment of functional capacity. In relation to the overall result, these were gender, age, marital status, level of education and type of residence. The impact of these variables has also been confirmed among other groups of Polish patients (Fidecki et al., 2022; Głowacka et al., 2017; Ulatowska et al., 2020). Interestingly, however, in the own research, people living alone achieved better results in all areas of the NOSGER scale. This may be due to the fact that single people have to be more resourceful and cannot count on, for example, loved ones who would do them in certain activities, not necessarily for health reasons, but for example because of the stereotype of an older adults as dependent on others, which exists in a given society.

On the Barthel scale, most of the respondents were classified as light (54.54%) or moderately heavy (43.63%). This percentage was higher than in other groups of older adults studied, for example those living in rural areas (Pillai and Paul, 2023). At the same time, this result was much lower than in patients with frailty or weakness syndrome (Haofen et al., 2023). The average number of points obtained by the respondents was similar to the result obtained, for example, by older adults hospitalized due to respiratory infections (Branche et al., 2022). An increase in the age of the subjects led to a decrease in their functional capacity. Similar results regarding the effect of age on functional capacity were obtained in older adults patients operated on for metastatic spine tumors (Kanda et al., 2023).

In the study group, the only demographic feature that had an impact on functional capacity was marital status. Married people had better functional capacity. In other studies, features that influenced the functional capacity of seniors included, for example, a lower level of education (Lopes Veira et al., 2023).

It is also important to remember that older adults cancer patients are less likely to be treated according to evidence-based recommendations. This may lead to a reduced sense of hope for recovery, development or intensification of symptoms of depression, which is rarely diagnosed in the group of seniors, especially those over 70 years of age (Goldzweig et al., 2022; Heidenreich et al., 2023). In the own study 66.36% of respondents were not at risk of developing depression. The average score of the respondents was 8.68. However, Bacik et al. (2023) in their research, they determined the incidence of depression in older adults people with cancer on the lower level. This result is similar to the prevalence of depressive symptoms among older people living in China (Sun et al., 2022). Interestingly, in some studies on the population of seniors without cancer, the percentage of people with depression may be much higher for example among seniors living in Turkey (Uysal and Poyraz, 2023) or Saudi Arabia (Alkhammash et al., 2022). This result may indirectly prove a change in the perception of the diagnosis of cancer from incurable to curable, thanks to, among others, ongoing campaigns promoting early diagnosis and constant progress in medicine. Loneliness may also be one of the factors leading to or worsening depression. Paradoxically, a diagnosis that requires frequent contacts with the health care system may provide these seniors not only with the appropriate amount of social contacts, but also be perceived by them as a source of support. However, social support itself may, in some cases, lead to a reduction in the severity of depression symptoms (Clausing et al., 2023). Women were more likely to suffer from depression among the respondents. This observation is confirmed by the results of other authors (Alkhammash et al., 2022). People who were not in a relationship and lived alone were also more susceptible to depression.

The results of the analysis of our own study based on the regression model confirmed that the NOSGER scale is a statistically significant variable explaining the geriatric depression scale score (higher NOSGER scores correspond to higher GDS scores). No such confirmation was obtained with regard to the Barthel scale.

The pilot research is not free from limitations. It is certainly the size of the study group and the cross-sectional nature of the research conducted. Due to the nature of the study, no calculations regarding sample size and margin of error were used, and data related to the characteristics of the tumor location, histopathological component of the tumor, and the extent of the disease were not analyzed. Due to the nature of the study, no calculations regarding sample size and margin of error were used, and data related to the characteristics of the tumor location, histopathological component of the tumor, and the extent of the disease were not analyzed. For this reason, it does not clearly answer the question about the impact of selected variables on functional capacity and the occurrence of depressive symptomatology among geriatric cancer patients. However, it can certainly be a starting element of a discussion on this topic in relation to this group of patients, which is rarely of interest to researchers. It seems reasonable to undertake further research, for example regarding the impact of the cancer treatment method used on functional capacity and depressive symptomatology in this group of patients. Research also proves the need to undertake various forms of interventions aimed at improving the functional capacity of seniors suffering from cancer, which should translate into an improvement in the quality of life of these people. An example of such activity may be, for example, dance classes (Nelson et al., 2023).

### Practical implications

The NOSGER can be a useful instrument for assessing patients in day-to-day geriatric practice. We believe that its use will help plan and implement holistic care for seniors, which may result in early detection of depression symptoms.

## 5 Conclusion

Geriatric assessment should also be included in the treatment of older adults cancer patients. Most of the subjects were fully functional. The surveyed seniors suffering from cancer had the greatest difficulties in the areas of moods and emotions as well as instrumental activities of everyday life. The occurrence of depression symptoms was found in over 30.00% of the respondents. Selected sociodemographic variables may influence both functional capacity and the occurrence of depressive symptomatology among these patients.

## Data Availability

The raw data supporting the conclusions of this article will be made available by the authors, without undue reservation.
